# Supplementary material for: Stable isotope (C, N, O, and H) study of a comprehensive set of feathers from two Setophaga citrina
Source: PLoS One. 2021 Jan 14;16(1):e0236536. doi: 10.1371/journal.pone.0236536 (PMC7808661; doi:10.1371/journal.pone.0236536)
Supplement: Attachment — Submitted filename: Response to Reviewers .docx [file pone.0236536.s002.docx]

Manuscript Revisions

**We thank both reviewers and the editors for their comments, the edits for which are down below.**

**Along with these comments, we made additional changes that we believe strengthen the arguments made in the manuscript. Firstly, we added additional discussion of Graves et al (2018), as this study also contains a feather set analysis similar to ours. We also clarified our methodology further and added justification for our addition of the rachis in our sample analysis, using Gordo (2020) and Bontempo et al (2014).**

**There are also corrections made to the values in Table 1, as some of the R^2^ were found to be incorrect during revisions. All values were checked, and the table now contains the correct values. None of the errors affect the argument of the main body.**

Reviewer 1

1. First, there needs to be more description about how the birds were stored prior to analysis. There appears to be some amount of relatively fast (ie, less than 2 weeks) H-isotope exchange between the keratin and water (Chamberlain et al., 1997). Chamberlain et al. (1997) conclude that only about 13% of the hydrogen is truly exchangeable; thus, the effect is likely to be small, but depends on the ambient water vapor dD and the dD values of the feathers when the warblers were collected. Given that there isn’t tremendous variability in dD between winter-time Central America, summertime along the Eastern Seaboard, and Houston air, I would further expect this effect to be small. Nevertheless, a simple calculation to show what the possible maximum effect of exchange would likely be (due to storage or delayed analysis) would help to clarify the meaning of the dD data.

**We appreciate reviewer’s comment, and we recognize that this is important for utilizing hydrogen isotopes as proxy for feather formation environments. As reviewer #1 states, feathers (mainly 
keratin) possess two groups of hydrogens: hydrogens bonded to carbons are largely non-exchangeable with the ambient environment after formation, whereas hydrogen bonded to nitrogen and oxygen can exchange with ambient environment. Therefore, only non-exchangeable hydrogen isotopes reflect the bird’s diet, water, and physiology at the time of feather growth.**

**The hydrogen isotope composition reported in our manuscript is of these non-exchangeable hydrogens, but unfortunately we did not state it clearly in our initial submission. We followed the comparative equilibration method (e.g. Wessnaar and Hobson, 2003, Soto et al 2017) in which samples and matrix-equivalent standard materials are treated identically―the exchangeable hydrogen isotopes are fully equilibrated with the laboratory environment before analysis. Because these lab air-equilibrated standards are the isotopic anchors for calibration, the measured isotopic variations are due to the non-exchangeable hydrogen isotope fraction of the sample feathers.**

**The procedure was as follows: We put weighed sample feathers and keratin standards CBS (Caribou Hoof  Standard) and KHS (Kudu Horn Standard) in closed tin capsules, open to the lab air, which allowed for re-equilibration of exchangeable hydrogen for both samples and standards with ambient air moisture. The exchange periods were 15 to 20 days. As Chamberlain et al. (1997) showed, isotopic exchange reaches completion at ~16 days. The equilibration processes took place during December to January (of the next year), with room temperature at ~23°C, RH~40 -50%. The hydrogen isotopic composition of lab air moisture was relatively stable, as demonstrated by the 1σ reproducibility of replicate CBS analyses: raw δD values were ~4‰ between batches in an  8-week period.**

**We have revised our manuscript with the addition of lines 109-118 explaining the above procedural details and added references.**

*“For δD values, 0.20 mg samples were* *measured along with Caribou Hoof standard (δD = -137‰) and Kudu Hood Standard (δD =-35‰). Weighed sample feathers and the standards CBS and KHS (both keratin standards) were put in loosely wrapped tin capsules, which allowed for re-equilibration of exchangeable hydrogen with ambient air moisture [20-21]. The equilibration took place from December to January (the next year) at a room temperature at ~23°C and a relative humidity of 40-50%. Under these conditions, complete isotopic equilibration should occur by the end of the 15 to 20-day period [22]. The hydrogen isotopic composition of lab air moisture appeared relatively stable during the winter based on the reproducibility of raw δD values for CBS between batches measured over an eight-week period (±4‰; 1σ). Because lab air-equilibrated keratin standards are also the isotopic anchors for calibration, the measured isotopic variations in the feathers are due to the non-exchangeable hydrogen isotope fraction.”*

2. The authors seem to somewhat “punt” and not actually dive into the d18O and dD data as much as one would like. First, it would be helpful to plot the feather dD against the feather d18O in order to clearly demonstrate that this relationship does not follow the global or really any meteoric water line. Second, the lack of a correspondence with an MWL doesn’t seem too surprising as Hobson et al. (2004) show that there is a poor correlation between feather d18O and local water d18O. Thus, it may be that only the dD is a tracer of local water. Here, though, I think the authors can again do a more thorough analysis than currently presented. Both Chamberlain et al. (1997) and Hobson et al. (2004) show that feather dD is depleted relative to meteoric water by about 10‰. In this study, that would suggest that the breeding ground meteoric water is in the range of -20 to -30‰. This does appear to be substantially higher than the dD values of meteoric waters in the Northeast and even down to the Potomac; it is, however, more in line with meteoric water dD values in the southeast (Kendall and Coplen, 2001). I don’t know what wintertime dD values are in Central America, but this can probably be approximated by searching [waterisotopes.org](http://waterisotopes.org/) (<https://wateriso.utah.edu/waterisotopes/pages/spatial_db/SPATIAL_DB.html>). I’d guess that something more specific can be said about the source of the water based upon the dD values even if no meteoric water line can be recovered from the feather d18O and dD.

**We appreciate the reviewer’s comments and recognize the importance of discussing the δD and δ^18^O in greater detail. As suggested, we have added a figure (Fig 4) with feather δD and δ^18^O plotted along with the local meteoric water lines of the greater United States, Southeastern United States, Northeastern United States, and Central America, using the suggested paper Kendall & Coplen (2001) and Lachniet and Patterson (2009). Discussion of this figure was added at line 197.**

**After discussion of this figure, we delved deeper into the discussion of the influence of diet and fluid intake on both δD and δ^18^O. A paragraph examining δD in greater detail was added in lines 209-220 and further explanation of the fractionation factor of δ^18^O was added in line 226. Discussion of the effects of diet using Wolf et al 2011 was also added at line 239.**

*“While the range of δD values for* *the full feather set showed some variability, excluding the body feathers shows relative homogeneity for the flight feathers. This contrasts with the findings of Graves et al. (2018) [15], which found δD variation within flight feathers for S. caerulescens. There are a few possible explanations for this discrepancy. One explanation could be methodology: Graves et al. (2018) [15] analyzed only the vane of the feathers, whereas both the vane and rachis were analyzed in this study. In addition, differences in the habitats, diets, and/or isotopic fractionation between the two species could also lead to detectable differences in isotopic trends. For example, while both species are insectivorous, S. caerulescens feeds over a wider diversity in structural height, from understory to lower canopy, whereas S. citrina primarily feeds in the understory. S. caerulescens also inhabit a wider altitude range (roughly 790–1,600 m in the summer months and close to sea level during wintering months) while S. citrina lives up to 1,100 m year-round; because δD values of precipitation decrease nonlinearly with altitude, the wider altitude range of S. caerulescens may also contribute to its larger isotopic variability [16, 30, 31].”*

*The magnitude of metabolic fractionation of ^18^O remains unclear, due to the larger number of sources and sinks for oxygen compared to hydrogen relevant for feather growth [1]. However, the δ^18^O values of the feathers may provide information about the molt location through a correlation with δ^18^O values in meteoric water [1, 10], similar to how δD values of the feathers can provide information about the molt location [1]. However, laboratory studies of Japanese quails and house sparrows show that the slope of the relationship between drinking water and δD can vary by species [33-34]. In this study, the relationship between δ^18^O and δD values for both samples neither followed the global meteoric water line (i.e., δD = 8*δ^18^O + 10)* *nor local meteoric water lines in the breeding or wintering regions, perhaps due in part to the narrow range of values observed (Fig. 4) [29]. Nevertheless, this finding is in agreement with the similarly weak correspondence between feather δ^18^O and δD values and local meteoric water lines observed in previous studies [1, 10, 33]. Broadly, this observation implies that δ^18^O and δD values of these feathers may be more strongly related to diet (as with δ^13^C and δ^15^N values) than with local hydrology. Isotopic enrichment factors for δ^18^O and δD isotopes in the S. citrina diet would presumably play a significant role in the isotopic signatures of their feathers. We note that Wolf et al. (2012) [35] found the relationship between diet and δD and δ^18^O values of feathers was weak in feathers for the Japanese quail, indicating that further study on these isotope systems is needed.*

3. The authors find that the d13C values are approximately -25‰ and suggest that there is an approximate enrichment of 3.5‰ from what the birds are eating, suggesting that the vegetation in the breeding grounds has a d13C of -28.5‰. However, my understanding is that these birds are insectivores, which likely additionally fractionate 13C (say 1 or 1.5‰), indicating that the base vegetation must be even lower in d13C (~ -29.5 or -30‰). This isn’t crazy low…instead, it might indicate that the breeding ground vegetation receives fairly high mean annual precipitation (> 1000 mm/year) (Kohn, 2010), perhaps further indicative that the breeding grounds are in the Southeast, or that the insects consumed by Hooded Warblers are targeting specific leaves that have lower d13C. Regardless, I’m not sure why the insect dietary fractionation of 13C can be excluded when calculating the original vegetation component.

**The previous lack of explanation for the discrimination factor for insects was an oversight in the original manuscript, so we appreciate the reviewer catching this issue. We have added a line explaining the discrimination factor in insects at line 184-190, using references from Chamberlain et al and McCutchan et al (2003).**

*Chamberlain et al. (1997) [22] found a similar δ^13^C enrichment of 3-4‰ in black throated blue warblers compared to local C3 plants. Isotopic compositions in insects can vary depending on species and their diet, but because most of the insects in the hooded warbler diet are herbivorous, we estimate the mean ^13^C enrichment of the insects relative to local plants using the general fractionation for whole consumers, i.e., ∆δ^13^C = 0.5 ± 0.13‰ [24]. Thus, the mean feather δ^13^C value of -25.0‰ suggests that the environment supporting S. citrina during molt had plants with mean δ^13^C values near -29‰, which is similar to the mean isotopic composition of C3 plants in temperate ecosystems (δ^13^C ~ -28.5‰) [25]*

**Reviewer 2**

Explain the used stable isotope systems and why they make sense for your study in more detail.
Consider including other studies of comprehensive sets of feathers and their results in context of your study. For example, the work by English et al. 2018 on museum specimens.

**We thank reviewer 2 for their comments and suggestions to the paper. We have included discussion on both English et al (2018) at line 25-28.**

*While there have been studies on the feathers of deceased birds, such as the use of museum specimens to examine the long-term evolution of stable-isotope composition of the Eastern Whip-poor-will, (Antrostomus vociferus), a complete set of feathers is rarely analyzed to study diet and migration [12].*

**and Graves et al (2018) from line 44- 50 in response to their suggestion to add more studies that include comprehensive feather sets.**

*Graves et al. (2018) [15] conducted an analysis of D/H ratios for 24-feather sets (9 primaries, 6 secondaries, 6 rectrices, and 3 patches of ventral contour feathers) from specimens of the black-throated blue warbler (Setophaga caerulescens). That study focused on the deuterium isotope composition in territorial male specimens to analyze variability generated during the complete annual prebasic molt. Results showed significant within-individual variations (i.e., δD values ranging from 12 to 60‰) across all pterolographic variables in the feather sets, as well as large interannual variability in both absolute δD values and their trends within feather classes.*

Line 57: Is it known why these birds died? Where they both male or female, or one of each? Would a sickness potentially cause changes to feeding behaviour and/or effect any of the used isotope systems?

**Specimens were found as window-killed samples on the Rice University grounds, with no indication of illness that could have affected the diet or feeding behavior before collection. Line 68 edited to clarify that samples were window-killed.**

*These specimens were window-kills ~~found by groundskeepers~~ on the campus of Rice University in Houston, Texas, USA (29.7° N, 95.4° W)*

Line 68: Analog to line 59, include the feather IDs used in Fig. 2 in brackets for those, who cannot print in colour.

**In line 78-82, which is the caption figure for Figure 2, IDs for all feathers were added to clarify the image.**

*Primary [P] and secondary [S] flight feathers are shown in red and blue respectively, along the right [RR] and left [RL] rectrices in green. The primary coverts [PC] are in yellow, the median coverts [MC] are in brown, and the greater coverts [GC] are in pink. The dorsal body feathers are also shown, with rump feathers shown in purple, back in orange, and throat in teal*

Line 83: Too long, shorten. Also, how often were the international standards analysed? Include n.

**Line 109 was edited for better wording.**

*For δD values, 0.20 mg samples were measured along with Caribou Hoof standard (δD = -137‰) and Kudu Hood Standard (δD =-35‰).*

**How often the standards were analyzed was added at line 121.**

*Standards for all analyses were measured six times for each sample run.*

Line 87: Why would you analyze your samples in duplicate or triplicate to evaluate external reproducibility? Should this not be evaluated using reference materials?

**Line 122-123 added to explain methodology of resampling**

*Some sample analyses were performed in duplicate or triplicate―depending on sample weight availability, particularly for any anomalous values (see below)―to evaluate external reproducibility.*

Line 97: Which one is it, n=2 or 3? If RL2 was replicated twice and S3 three times, then it would be better to write this as: … of the anomalous feathers RL2 and S3 (n= 2 and 3, respectively) suggest…

**Samples were analyzed in triplicates only if there was enough sample left to do so, as some of the feathers were considerably smaller than others. Both comments relate to this methodology, so explanation of this was added at Line 122 and line 131.**

*Some sample analyses were performed in duplicate or triplicate (depending on sample weight availability), particularly for any anomalous values, to evaluate external reproducibility.*

*Replicate analyses of these anomalous feathers (n = 2 or 3 depending on available sample weight) suggest that the anomalies are analytically robust.*

Line 106: Here and elsewhere, when giving mean values and the p-value, please include n.

**N-values were included wherever mentioned throughout Results sections.**

Line 109: The error range of your mean values when comparing the 2017 vs 2018 bird, are app. equal (d15N) or even higher (dD) than the mean value – this error range seems to undermine the usefulness of a mean value. Perhaps reconsider what this range and the inferred difference between bird 2017 and 2018 means.

**Error ranges were changed to represent standard error of the mean instead of previous value to give a more accurate view of the values stated in line 143-144.**

*The mean δ^15^N, δ^18^O, and* *δD values of the 2018 bird were 0.8 ± 0.1‰ higher, 1.0 ± 0.1‰ lower, and 1.8 ± 0.6‰ higher (1 s.e.m.), respectively, than those of the 2017 bird.*

Line 119: It is interesting that r2 for d13C are more often in disagreement between bird 2017 and 2018; particularly S1 to P9, S1 to S7, and RL1 to RL6 show a high r2 for bird 2017, while the r2 values show no correlation for bird 2018. When you highlight one high correlation for d18O, RR1 to RR6 for bird 2017, consider evaluating Table 1 in more detail.

**A line was added at Line 155 to discuss this observation**

*Similarly, for δ^13^C, high correlation in one sample for the flight feathers and left rectrices were not replicated in the other sample.*

Line 140: Expand on your reasoning for this.

**Line added at line 175 to clarify reasoning.**

*It is not clear if these systematic differences are related to slightly different isotopic fractionation during the growth of body feathers or slightly different molt locations for the body feathers (i.e., in the more southerly wintering grounds). Both possibilities should be explored in more detail with S. citrina samples from a known molt location.*

Line 165: What do you mean with ‘rare’ oxygen and hydrogen isotopes? It would be better to refer to, e.g., C-13 as less abundant.

**Wording in line 238 changed to** **δ^18^O and δD to clarify meaning.**

*Isotopic enrichment factors for δ^18^O and δD ~~rare oxygen and hydrogen isotopes~~ in the S. citrina diet may also play a significant role in the isotopic signatures of their feathers.*
